# The study of the rs9939609 FTO gene polymorphism in association with obesity and the management of obesity in a Romanian cohort

**Published:** 2015

**Authors:** RI Ursu, C Badiu, N Cucu, GF Ursu, I Craciunescu, E Severin

**Affiliations:** * Department of Medical Genetics, ”Carol Davila” University of Medicine and Pharmacy, Faculty of Medicine, Bucharest, Romania; ** ”C.I. Parhon” National Institute of Endocrinology, Bucharest, Romania; *** Genetics Department, Faculty of Biology, University of Bucharest, Romania; **** Epigenetics Center/ ITA-BINNOTECH -NIBS, Bucharest, Romania; ***** "Agrippa Ionescu” Military Emergency Hospital, Cardiovascular Clinic, Bucharest, Balotesti, Romania; ****** Department of Genetics, ”Carol Davila” University of Medicine and Pharmacy, Faculty of Dental Medicine, Bucharest, Romania

**Keywords:** obesity, FTO, rs9939609, association

## Abstract

The incidence of obesity especially in Romanian population is presently escalating as a major nutrition and health problem. Clinicians aided by scientists are engaged in research approaches that include heredity aspects linked with behavior, education, applied nutrition studies and clinical therapies in order to prevent, control and reverse obesity. The common goal is to identify areas of basic and clinical research to understand aspects of human biology that may be considered as obesogenic. Regarding these approaches, recent discoveries in genetics, epigenetics and functional genomics, based on advancing technologies, are tools employed to prevent and treat obesity. The purpose of this article is to present the current knowledge of key components of the FTO gene role in the obesogenic system that links genetic, epigenetic and environmental, lifestyle/ diet nutritional and behavioral components and to describe the results obtained by genotyping and interviewing relevant selected groups of Romanian population.

FTO rs9939609 genotyping was performed on a Romanian study group of 53 subjects (30 obese, 23 normal). Results have been analyzed in association with obesity parameters and comorbidities in order to identify this polymorphism’s effect on body mass in our Caucasian cohort. At the same time, personal history of the subjects in correlation with the FTO genotypes provided important information on the FTO gene’s influence on the feeding behavior and food selection of these individuals.

In conclusion, the FTO rs9939609 polymorphism has been identified as a common gene variant in our Romanian Caucasian cohort, proving a high association with all the parameters of obesity and obesity comorbidities. The adherence to a Mediterranean diet is benefic for subjects with genetic predisposition for this disorder as long as it is kept for a long period of time along with sustained physical exercise. Association studies are an extremely important tool in understanding the hunger-satiety pathway, providing information on the relation between obesity-related genes, gene expression and behavior.

## Introduction

Obesity is one of the most common disorders worldwide, affecting individuals of all ages, races, sexes, social and cultural environments and providing the predisposition to a series of severe complications and co-morbidities, such as type II diabetes mellitus, hepatic steatosis, hypertension, cardiovascular disease, stroke, atherosclerosis, embolism, cancer [**1**-**3**]. There are three known types of genetic obesity – monogenic (syndromic) obesity, pleiotropic (syndromic) obesity and polygenic (common, multifactorial, non-syndromic) obesity [**4**-**7**]. While the syndromic obesity (monogenic and pleiotropic) is represented by rare, singled-out cases of complex genetic syndromes in which obesity is the or one of the main affections, caused by large gene or chromosome abnormalities, polygenic obesity stands for more than 80% of the cases worldwide, being by far the most common type [**4**,**5**,**8**]. 

Monogenic obesity is represented by genetic syndromes in which obesity is the central and most often sole clinical element, produced by mutations in a single gene which is involved in the hunger-satiety pathway [**4**,**6**,**9**]. Genetic approaches identified so far a number of single causal mutated genes of genetic obesity syndromes, the most frequently reported ones being leptin (LEP), leptin receptor (LEP-R), melanocortin 4 receptor (MC4R), proopiomelanocortin (POMC), prolactin (PRL), Niemann-Pick disease type C1 gene (NPC1), V-maf musculoaponeurotic fibrosarcoma oncogene homolog (MAF), and others [**8**-**10**].

Pleiotropic obesity syndromes are complex multisystemic genetic disorders caused by complex multigene mutations or epigenetic modifications that do not obey to Mendelian rules, or even chromosomal alterations (deletions, translocations, duplications, inversions, etc.). These anomalies produce a series of clinical features, one of them being obesity. Examples of these types of syndromes are Prader-Willi syndrome (PWS), Bardet-Biedl syndrome (BBS), Albright hereditary osteodistrophy (PHP1A), Alstrom syndrome (ALMS), Cohen syndrome (COH1), Fragile X syndrome (FXS), Borjeson–Forssman–Lehmann syndrome (BFLS), Simpson-Golabi-Behmel type 2 syndrome (BFLS) and others. There have been more than 30 pleiotropic obesity syndromes described so far, all of them being rare genetic disorders [**11**,**12**].

The common type of obesity is nevertheless the polygenic one. Obesity is the result of an unbalance between the intake and expenditure of a genetic predisposition (produced by a number of gene variants) which may transform into the actual disease if the environmental factors are met. There are a number of factors that may cause a predisposition to turn into obesity: behavioral, psychological, social, cultural, religious, geographic, etc. [**3**,**4**,**13**,**14**]. 

In 2007, the first gene described in association with the common obesity was the fat mass and obesity associated gene (FTO) [**15**,**16**]. Since then, there have been over 60 chromosome locations with more than 240 gene polymorphisms identified of being correlated with a higher weight gain. Finding the genes and mutations responsible for the obesity pathogenesis has become the focal point of research for this disease, as they will describe, either the predisposition or the development of this disorder depending on the type of mutation and number of genes involved [**14**,**17**-**20**].

There are two types of studies in the genetic research of obesity – candidate gene studies and genome wide association studies (GWAS). The candidate gene studies have been the first ones essayed and they aim at the monogenic types of obesity and partially at the pleiotropic types. The candidate gene studies identified leptin to be strongly correlated with obesity, however multiple other genes responsible for monogenic obesity syndromes have been described through candidate gene studies (POMC, LEP-R, MC4R, PRL, etc.) [**8**-**10**]. Presently, a map of genes causing obesity in pleiotropic syndromes (SNRPN gene for the obesity in PWS, GNAS1 gene in PHP1A, ALMS1 gene in ALMS, FMR1 gene in FXS, and others), has been established [**11**,**12**]. However, the candidate gene studies cannot determine the large number of genes and single nuclear polymorphisms (SNPs) involved in the common type of obesity.

Genome-wide association studies can afford to approach at least a few hundred SNPs at a time and then correlate the findings to build gene association maps. As stated before, in the field of obesity, the first gene that proved to be correlated with the polygenic type was FTO. 

Our research was centered on the FTO rs9939609 gene polymorphism because the FTO and MC4R genes are the most frequently associated causal markers of polygenic obesity, the rs9939609 SNP being the most commonly described in correlation with this disorder and different metabolic syndrome characteristics (obesity, BMI, abdominal circumference, weight, WHR (Waist/ Hip Ratio), body fat percentage) [**18**,**19**,**21**-**25**]. FTO mRNA is expressed in hypothalamic nuclei and is involved in energy balance regulation in mice [**23**-**25**]. Recent studies indicate that the FTO mRNA is highly regulated by fasting, feeding, eating behavior in general and environment, revealing an important link between the FTO gene and its chromosome address (16q12.2) and the hunger sensation [**23**-**26**]. Intron 1 of the gene is the only one associated with obesity, multiple FTO gene variants being at that location which is correlated to this disorder: rs9939609, rs17817449, rs1421085, rs9930506, rs8050136, rs9751812, rs9751812 and other 25 validated SNPs [**23**-**26**]. 

## Materials and methods

**Patient selection.***DNA genotyping* was performed on a total of *53 subjects* (30 adults and 23 children), 34.28 ± 24.19, (8–88) years old, 50.94% male, separated into 2 *study groups*: a). *Normal weight group/ control group* – 19 subjects (9 adults, 10 children), 34.28 ± 24.4, (8–86) years old, 52.63% male; and b). *Obese group* – 34 subjects (21 adults, 13 children), 37.41 ± 23.85, (9–88) years old, 50% male.

The biological material used was DNA extracted from *saliva* and *blood*. All the selected subjects in the study have given their *written informed consent* for the procedures and the use of their biological material and data and the *study has been approved by the Bioethics Committee of the National Commission of Romania for UNESCO*.

Study inclusion and exclusion criteria. The *main criteria for the subjects’ inclusion in the study was the Body Mass Index (BMI)*, as it follows: 1). normal weight: 18.5–24.9 kg/ m2; 2). overweight: 25–29.9 kg/ m2 and 3). obese: > 30 kg/ m2. All the individuals belonging to the normal group (controls) were selected with BMI values below 24.9 kg/ m2, while subjects from the obese group were selected with BMI values higher than 24.9 kg/ m2. *Abdominal (waist) circumference and body weight (mass)* were also taken into account when selecting the 2 groups, as they are direct indicators of abdominal obesity. Subjects on treatment (or diet) for obesity and obesity associated disorders were excluded from the study.

**Medical investigations (clinical/ lab work)**

***Anamnesis*** revealed the presence of abdominal obesity, obesity comorbidities and obesogenic risk factors *(personal history)* as well as the *family (heredocolateral)* obesity *history*.

***Physical measurements*** performed for association studies: waist (abdominal) circumference (cm), weight (kg), height (cm), BMI (kg/ cm2, by the formula weight/ height2), basal metabolism ratio (BMR, calories, by the metric BMR formula), total daily caloric needs (calories, by the Harris Benedict Equation), body fat percentage (%, by the metric Body Fat Formula) and body fat mass (kg, by the formula body fat percentage*weight), blood pressure (mmHg, in ortho-/ and clinostatism, by using the Yton-Gima manual aneroid sphygmo-manometer).

The ***biochemical parameters*** investigated in this study were the following: fasting glucose and dueing OGTT (mg/ dl), lipids levels – total cholesterol, HDL-cholesterol, LDL-cholesterol, triglycerides (mg/ dl), thyroid stimulating hormone – TSH (mU/ L) and transaminases levels – GOT (AST), GPT (ALT) (U/ l). All the evaluations were performed on a Roche analyzer, COBAS INTEGRA system, by using the ELISA photometric method.

For the association of the studies with the FTO rs9939609 gene polymorphism, a number of *obesity comorbidities* found in the obese subjects were evaluated along with the physical measurements and the biochemical parameters: *hypertension, diabetes mellitus, thyroid affection, hepatic steatosis, dyslipidemia, hypercholesterolemia, hypertriglyceridemia, metabolic syndrome, basal metabolism changes*.

**DNA genotyping method**

The FTO rs9939609 polymorphism genotyping was performed on an Arkray I-Densy 5320 system by using the High Resolution Melting Quenching Probe’s temperature (HRM-QP) analysis. Saliva for DNA extraction was collected and isolated by using IsoHelix Buccal Swabs and Isolation Kits.

**Statistical interpretation and association analysis of the results**

The data obtained through DNA genotyping were compared and analyzed in correlation with the clinical and paraclinical measurements by using the *statistics SPSS software* (version 13.0) and the *Microsoft Excel Analyse–it Tool Pack 1.71*. Means comparisons, Spearman and Pearson correlations, ANOVA analysis, Tamhane’s T2, multiple comparisons, t-test, descriptive analysis (Frequencies, Descriptives, Ratios, Crosstabs), Fisher Exact Probability test, bivariate correlations, Krustal-Wallis test, cross-variations, mediation analysis, linear regression) were statistical-mathematical methods used for the results interpretation and association studies. 

## Results

FTO rs993969 genotyping was performed on two individual groups selected based on specific criteria associated with obesity diagnosis and comorbidities. Both data obtained from the genotyping and clinical and paraclinical investigations were then correlated separately and collectively to identify the association between the rs9939609 polymorphism of the gene and obesity, obesity parameters and obesity comorbidities. All the measured parameters (age, gender, body parameters, transaminases, TSH and lipids levels, blood pressure levels) were included in the association analysis with the mutant T allele of the gene and the 3 different FTO genotypes (homozygous mutant, HmMut – A/ A, heterozygous, Ht – A/ T, homozygous wildtype, HmWt – T/ T). 

**Differences between the two selected individual groups**

***Clinical and paraclinical parameters***

The 2 study groups were statistically different regarding the following parameters: weight (p=0.000, t-test analysis), abdominal circumference (p=0.000, t-test), BMI (p=0.000, t-test), basal metabolic rate (p=0.000, t-test analysis) and total caloric needs (p=0.000, t-test), body fat percentage (p=0.000, t-test), mass (p=0.000, t-test) and excess (p=0.000, t-test), total cholesterol (p=0.000, t-test), LDL-cholesterol (p=0.002, t-test), HDL-cholesterol (p=0.032, t-test analysis) and triglycerides levels (p=0.003, t-test), blood pressure levels (systolic p=0.000, t-test; diastolic p=0.001, t-test), fasting glucose levels (p=0.003, t-test), GOT (AST) (p=0.004, t-test) and GPT (ALT) (p=0.000, t-test analysis) levels. The only variables which were not statistically different between the 2 groups were height (p=0.363, t-test), age (p=0.211, t-test), sex (p=0.858, t-test) and TSH levels (p=0.204, t-test). 

***Obesity comorbidities***

As mentioned in the previous paragraphs, all the obesity associated disorders were found only in the obese subjects, so the difference between the 2 groups in obesity comorbidities was strongly statistically significant: obesity level (overweight/ obesity class I/II/III) (p=0.000, t-test), glucose metabolism status (impaired glucose tolerance/ diabetes mellitus) (p=0,000, t-test), hypertension (p=0.000, t-test), dyslipidemia (p=0.000, t-test), hypercholesterolemia (p=0.000, t-test), hypertriglyceridemia (p=0.000, t-test), metabolic syndrome (p=0.000, t-test), hepatic steatosis (p=0.008, t-test). No statistical difference was found between the 2 groups and hypothyroidism (p=0.125, t-test) or diabetes mellitus (p=0.082, t-test), although no subjects from the control group had any of these disorders.

**FTO rs9939609 association analysis**

***FTO rs9939609 genotypes and mutant (A) allele frequencies***

Our previous studies on adults and children separately revealed a strong correlation when comparing the 3 FTO genotypes between the obese and normal groups. In the adults study (30 adults, 50% male; 21 obese, BMI = 37.98 ± 7.46 kg/m2/9 normal, BMI=22.5 ±1.48 kg/ m2), the correlation between the mutant FTO allele and the two groups was statistically significant (p=0.000, ANOVA test). The same association was identified in the children study (23 children, 52.2% male; 13 obese, BMI=33.4 ± 6.73 kg/m2/10 normal, BMI = 22.47 ± 0.76 kg/ m2). Although not as strong as in the adults study, the difference between the 2 children groups was significant when comparing the 3 FTO genotypes (p=0.04, ANOVA test). 

As expected, the presence of the A allele showed a strong statistical correlation with obesity in this extended study (comprising both children and adults), with a significant difference between the 2 study groups (p=0.000, ANOVA test). The differences between the T/ T genotype and any of the other 2 (T/ A, A/ A) containing the mutant allele were also statistically significant (p=0.006, TT/ TA; p=0.000, TT/ AA, ANOVA Tamhane test), the predisposition for obesity and obesity related comorbidities increasing with the presence of each A allele. 

***Correlation between the FTO rs9939609 genotypes and clinical/ paraclinical parameters and obesity comorbidities***.

Results from the FTO rs9939609 gene polymorphism association analysis are listed in **Table 1**. 

**Table 1:** Differences between the 3 FTO genotypes and Clinical/ paraclinical parameters and obesity associated disorders

*MEASURE*	**HmWt** (15/53, 28.3%)	**Ht** (20/53, 37.74%)	**HmMut** (18/53, 33.96%)	P value ANOVA
*Weight (kg)*	60.20 ± 15.76 (36.00 – 87.00)	81.40 ± 28.52 (35.00 – 130.00)	112.28 ± 35.16 (65.0 – 191.0)	0.000
*Abdominal circumrence (cm)*	77.27 ± 11.63 (62.00 – 107.0)	95.45 ± 30.13 (59.00 – 199.00)	111.67 ± 30.01 (68.0 – 180.0)	0.002
*BMI (kg/cm2)*	23.37 ± 3.98 (19.20 – 32.70)	30.33 ± 7.52 (20.03 – 45.90)	37.88 ± 9.43 (21.90 – 55.10)	0.000
*BMR (calories)*	1500.40 ± 208.23 (1173.00 – 1903.60)	1706.19 ± 379.74 (1189.20 – 2495.40)	1983.01 ± 568.30 (1135.40 – 3206.10)	0.007
*Total caloric needs (calories)*	2063.06 ± 286.31 (1612.88 – 2617.45)	2346.02 ± 522.14 (1635.15 – 3431.18)	2726.64 ± 781.41 (1561.18 – 4408.39)	0.007
*Body.fat.percentage (%)*	14.55 ± 9.23 (0.47 – 33.72)	19.08 ± 10.76 (-5.64 – 34.30)	27.87 ± 9.67 (6.63 – 50.41)	0.001
*Body fat mass (kg)*	9.49 ± 7.64 (0.17 – 29.34)	17.97 ± 12.14 (-1.97 – 41.38)	32.14 ± 18.53 (4.71 – 76.62)	0.000
*Excess body fat mass (kg)*	3.48 ± 6.61 (-4.26 – 20.64)	9.83 ± 10.03 (-5.47 – 32.38)	21.05 ± 15.66 (-2.39 – 61.42)	0.000
*Fasting glucose (mg/dl)*	87.66 ± 6.88 (73.00 – 101.00)	94.40 ± 10.90 ± 75.00 – 112.00	117.22 ± 26.59 (90.0 – 175.0)	0.000
*Systolic blood pressure (mmHg)*	113.73 ± 4.36 (105.0 – 120.0)	136.5 ± 23.85 (110.0 –193.0)	152.16 ± 30.6 (110.0 – 210.0)	0.000
*Diastolic blood pressure (mmHg)*	73.40 ± 4.06 (67.00 – 80.00)	87.20 ± 18.77 (68.0 – 146.0)	86.11 ± 15.99 (60.0 – 130.00)	0.020
*TSH (µU/ml)*	3.22 ± 1.16 (1.74 – 6.42)	3.67 ± 2.52 (1.50 – 12.30)	3.19 ± 1.76 (1.10 – 7.52)	0.700
*Total cholesterol (mg/dl)*	167.33 ± 27.56 (133 – 205)	194.75 ± 48.00 (129 – 290)	220.67 ± 40.34 (135 – 303)	0.002
*LDL-cholesterol (mg/dl)*	105.27 ± 21.71 (72 – 150)	124.65 ± 36.46 (69 – 200)	152.06 ± 33.23 (80 – 200)	0.000
*HDL-cholesterol (mg/dl)*	37.93 ± 10.97 (27 – 73)	42.45 ± 19.75 (24 – 120)	39.66 ± 9.64 (30 – 64)	0.653
*Triglycerides (mg/dl)*	108.67 ± 39.50 (36 – 208)	127.60 ± 51.02 (58 – 220)	164.28 ± 39.10 (103 – 225)	0.002
*AST (GOT) (U/l)*	25.46 ± 8.36 (13.00 – 41.00)	30.20 ± 18.54 (10.0 – 72.00)	30.87 ± 19.34 (10.00 – 85.00)	0.608
*ALT (GPT) (U/l)*	27.46 ± 10.22 (12.0 – 49.00)	47.15 ± 41.49 (12.0 – 131.0)	42.74 ± 38.19 (12.0 – 145.00)	0.235
Obesity* (N) (34/53, 65.15%)	3/15, 20 %	14/20, 70 %	17/18, 94.44 %	0.000
Hyperglicemia** (N) (31/53, 58.49%)	1/15, 6.67 %	16/20, 80 %	14/18, 77.78 %	0.000
*Diabetes mellitus (N) *(9/53, 16.98%)	0/15, 0 %	1/20, 5 %	8/14, 44.44 %	0.066
*Hypertension (N)*(21/53, 39.62%)	0/15, 0 %	10/20, 50 %	11/18, 61.11	0.000
*Hypothyroidism (N) *(4/53, 7.55%)	0/15, 0 %	3/20, 15 %	1/18, 5.56 %	0.243
*Dyslipidemia (N) *(26/53, 49.05%)	3/15, 20 %	9/20, 45 %	14/18, 77.78 %	0.003
*Hypercolesterolemia (N) *(26/53, 49.05%)	3/15, 20 %	9/20, 45 %	14/18, 77.78 %	0.003
*Hypertriglyceridemia (N) *(19/53, 35.85%)	1/15, 6.67 %	6/20, 30 %	12/18, 66.67 %	0.001
*Metabolic syndrome (N) *(23/53, 43.39%)	2/15, 13.33 %	8/20, 40 %	13/18, 72.22 %	0.002
*Hepatic steatosis (N) *(10/53, 18.87%)	0/15, 0 %	5/20, 25 %	5/18, 27.78 %	0.088
N = no. of cases;* overweight/ obese; ** impaired glucose tolerance/ diabeste mellitus				

## Discussion

***FTO rs9939609 genotypes and mutant (A) allele frequencies***

Out of the total of 53 subjects from both study groups, the *homozygous wildtype* (T/ T) genotype was identified in 15 individuals (28.3%), the *heterozygous* (T/ A) in 20 (37.7%) and the *homozygous mutant* (A/ A) in 18 (34%). The mutant A allele was identified in 38 of the 53 subjects (71.7%), 13.7% higher than reported for the Caucasian population (58%) [**27**], making the FTO rs9939609 polymorphism a possible common gene variant for the Caucasian Romanian population, although the number of individuals in this study was not big enough for such assumptions. Our previous studies revealed high frequency rates for the mutant A allele both in adults (83.33%) and children (56.52%). The difference between these percentages may be an indicator that the A allele is more of an indicator for obesity in adults than in children for our Romanian cohort. 

The extended frequency of the A allele is 56/ 106 alleles (52.83%), meaning that 56 out of the 106 subjects’ parents carried at least one mutant FTO rs9939609 allele which was passed on to their descendants. In this respect, the study of a number of obesity-affected families from our study groups was essayed with significant results on the inheritance of the FTO rs9939609 obesity predisposition. However, the results of this study are yet to be published.

***Study results and further management of the subjects***

The results of our study revealed important links between the FTO genetic predisposition for obesity and the development of the disorder itself. These findings suggested that this genetic predisposition may be kept under control only if it is known in due time (neo-/ postnatal) or if the lifestyle of the subjects includes high physical exercise and a constant diet. 

A number of subjects included in the normal groups were discovered to carry the homozygous mutant (A/ A) FTO genotype, thus a high genetic obesity predisposition. However, given their lifestyles (professional athletes, individuals always concerned with their diet, army officers, etc.), it was easy to understand the lack of body fat excess against the highly expressed genetic marker. What should also be mentioned is that these respective subjects kept their lifestyles constant throughout all their lives and had no personal history of obesity whatsoever.

On the other hand, a number of subjects from the obese groups revealed a mutant wildtype (T/ T) genotype, presenting no FTO rs9939609 predisposition. This may mean that other genetic, obesity-related markers were present in these subjects producing the predisposition to develop obesity. 

Some of the adults in the obese group were retired professional athletes, who maintained an optimal physical shape during their professional careers, but after retirement, they rapidly developed severe obesity. The highest values of weight were obtained from individuals with this personal history. All these subjects were carrying at least 1 mutant (A) FTO allele but against their genetic predisposition, through intense physical exercise, they did not develop obesity while performing their professions. However, once the physical activity was reduced or mostly stopped and athletics-specific diet replaced with normal modern day alimentation, the genetic predisposition took over and cumulated with the increased caloric needs, and, due to the highly developed muscle system, generated severe obesity.

A number of studies highlighted the benefits of the Mediterranean diet plan in individuals carrying a genetic predisposition for developing obesity in general [**28**,**29**] or the specific FTO predisposition [**30**,**31**]. Indeed, part of the subjects referred to in the previous paragraph, were guided by the nutritionist to adopt the Mediterranean diet plan. The results were benefic and fast, after 6-months theses, the subjects losing between 47.15% and 66.75% of their body fat mass (19.8–51.1 kg, 70.37-89.56% of their excess body fat mass). However, with no exception, when the diet plan was stopped, diet cancelled and physical exercise reduced due to the normal sedentary modern lifestyle, all of these subjects gained back 14.7–43.2 kg in the same or smaller amount of time and the tendency for all of them was to gain more.

These cases came upon our knowledge to suggest that both the genetic predisposition and diet programmes are relevant when certain factors are met. The genetic obesity predisposition (FTO rs9939609 gene variant in this case) may develop into the disease but not necessarily. If the subjects are aware of their genetic inheritance and adopt specific proved diets or regular physical exercise they may cancel their predisposition and never develop obesity. However, diets and athletic activities are effective if maintained and included into the lifestyle of the individuals. 

When performing the anamnesis, poor eating and sedentary behavior were the main causes of obesity in our subjects. Food selection is an important factor, as the speed of the modern day life rarely allows individuals to be able to carefully choose their food and have a constant meal plan. Eating whatever, whenever, just to answer a sensation often misinterpreted as hunger, has given obesity the epidemic status. Hunger sensation comes as a reaction to the lack or need of energy (calories), but it may also be the result of emotional stress or usual behavior. Association studies are useful especially in these types of situations, when a great number of genetic markers are involved in giving the apparently same sensation – hunger. Understanding what makes us want to eat is an important piece in the signaling obesity puzzle; a piece we are yet to fully understand. 

## Conclusions

1. The rs9939609 polymorphism is a highly frequent gene variant in our study groups, with a high incidence of the mutant A allele.

2. The A allele has been found statistically correlated with all the obesity parameters (weight, abdominal circumference, BMI, body fat mass, percentage and excess, basal metabolic rate) and most of the biochemical and physical parameters (systolic and diastolic blood pressure, total cholesterol, LDL-cholesterol and triglycerides levels).

3. No correlation has been found between the A allele and HDL-cholesterol, TSH, ALT and AST levels.

4. The A allele has been identified in statistical association with obesity-related disorders found in our subjects (altered glucose metabolism status, hypertension, dyslipidemia, hypercholesterolemia, hypertriglyceridemia, metabolic syndrome).

5. No association has been found between the FTO genotypes and diabetes mellitus, hepatic steatosis and hypothyroidism in our groups.

6. However, the mutant FTO A allele was found more in correlation with obesity in adults than in children, both being statistically significant.

7. The genetic FTO rs9939609 obesity predisposition can be cancelled with constant life-long physical exercise and diet.

8. Mediterranean diet is useful in our Caucasian subjects to decrease the body fat mass as long as it is followed for a long period of time or as a lifestyle.

9. Eating behavior, poor meal selection, emotional stress are important causes of developing obesity in our subjects and setting them straight should be the starting point of every diet.

10. Genome wide association studies might create the links needed for clinicians to understand the full process of obesity development and hunger signaling in order to be able to adopt healthy screening programmes and suitable diet plans. 

## References

[1] Turconi G, Cena H (2013). Epidemiology of obesity. In: Bagchi D, Preuss HG. Obesity. Epidemiology, Pathophysiology and Prevention.

[2] Ursu GF (2009). Sindromul X metabolic (X metabolic syndrome). PhD thesis.

[3] Balaban G, Silva G (2004). Protective effect of breastfeeding against childhood obesity. J Pediatr.

[4] Farooqi IS, O’Rahilly S (2006). Genetics of Obesity in Humans. Endocrine Reviews.

[5] Hinney A, Vogel CIG, Hebebrand J (2010). From monogenic to polygenic obesity: recent advances. Eur Child Adolesc Psychiatry.

[6] Bircan I (2009). Genetics of Obesity. J Clin Res Ped Endo.

[7] Choquet H, Meyre D (2011). Molecular Basis of Obesity: Current Status and Future Prospects. Current Genomics.

[8] Herrera BM, Lindgren CM (2010). The Genetics of Obesity. Curr Diab Rep.

[9] Farooqi IS, O’Rahilly S (2004). Monogenic Human Obesity Syndromes. Recent Progress in Hormone Research.

[10] Meyre D, Froguel P (2010). Monogenic obesity. In: Freemark M. Pediatric obesity. Etiology, pathogenesis and treatment.

[11] Haqq AM (2010). Syndromic obesity. In: Freemark M. Pediatric obesity. Etiology, pathogenesis and treatment.

[12] Goldstone AP, Beales PL (2008). Genetic obesity syndromes. Front Horm Res.

[13] Hinney A, Hebebrand J (2010). Polygenic obesity. In: Freemark M. Pediatric obesity. Etiology, pathogenesis and treatment.

[14] Ursu RI (2013). Obesity, a gene review. Bulletin of the Transylvania University of Brasov, Series VI – Medical Sciences.

[15] Dina CD, Meyre S, Gallina E, Durand A, Korner P, Jacobson LM, Carlsson W, Kiess V, Vatin C, Lecoeur, Delplanque J, Vaillant E, Pattou P, Ruiz J, Weill J, Levy-Marchal C, Horber F, Potoczna N, Hercberg S, Le Stunff C, Bougnères P, Kovacs P, Marre M, Balkau B, Cauchi S, Chèvre JC, Froguel P (2007). Variation in FTO contributes to childhood obesity and severe adult obesity. Nat Genet.

[16] Gerken T, Girard CA, Tung YC, Webby CJ, Saudek V, Hewitson KS, Yeo GS, McDonough MA, Cunliffe S, McNeill LA, Galvanovskis J, Rorsman P, Robins P, Prieur X, Coll AP, Ma M, Jovanovic Z, Farooqi IS, Sedgwick B, Barroso I, Lindahl T, Ponting CP, Ashcroft FM, O'Rahilly S, Schofield CJ (2007). The obesity-associated FTO gene encodes a 2-oxoglutarate-dependent nucleic acid demethylase. Science.

[17] Zhao J, Bradfield JP, Li M, Wang K, Zhang H, Kim CE, Annaiah K, Glessner JT, Thomas K, Garris M, Frackelton EC, Otieno FG, Shaner JL, Smith RM, Chiavacci RM, Berkowitz RI, Hakonarson H, Grant SF (2009). The role of obesity-associated loci identified in genome wide association studies in the determination of pediatric BMI. Obesity.

[18] Loos RJF (2012). Genetics determinants of adiposity. In: Symonds ME. Adipose Tissue Biology.

[19] Lu Y, Loos RJF (2013). Obesity genomics: assessing the transferability of susceptibility loci across diverse populations. Genome Medicine.

[20] Pérusse L, Bouchard C (2000). Gene diet interactions in obesity. Am J Clin Nutr.

[21] Ortega-Azorín C, Sorlí JV, Asensio EM, Coltell O, Martínez-González MA, Salas-Salvadó J, Covas MI, Arós F, Lapetra J, Serra-Majem L, Gómez-Gracia E, Fiol M, Sáez-Torm G, Pintó X, Muñoz MA, Ros E, Ordovás JM, Estruch R, Corella D (2012). Associations of the FTO rs9939609 and the MC4R rs17782313 polymorphisms with type 2 diabetes are modulated by diet, being higher when adherence to the Mediterranean diet pattern is low. Cardiovascular Diabetolog.

[22] FTO rs9939609. http://snpedia.com/index.php/Rs9939609.

[23] FTO gene. http://omim.org/entry/610966?search=fto&highlight=fto.

[24] FTO gene. http://www.genecards.org/cgi-bin/carddisp.pl?gene=FTO&search=c20465d8c96dce19df855790c2b2e1e0.

[25] FTO gene. http://www.ncbi.nlm.nih.gov/gene/79068.

[26] FTO gene. http://ghr.nlm.nih.gov/gene/FTO.

[27] FTO rs9939609 population genetics. http://www.ensembl.org/Homo_sapiens/Variation/Population?db=core;r=16:53786115-53787115;v=rs9939609;vdb=variation;vf=6529447.

[28] Funtikova AN, Benítez-Arciniega AA, Gomez SF, Fitó M, Elosua R, Schröder H (2014). Mediterranean diet impact on changes in abdominal fat and 10-year incidence of abdominal obesity in a Spanish population. Br J Nutr.

[29] Tognon G, Moreno LA, Mouratidou T, Veidebaum T, Molnár D, Russo P, Siani A, Akhandaf Y, Krogh V, Tornaritis M, Börnhorst C, Hebestreit A, Pigeot I, Lissner L (2014). Adherence to a Mediterranean-like dietary pattern in children from eight European countries. The IDEFICS study. International Journal of Obesity.

[30] Razquin C, Martinez JR, Martinez-Gonzalez MA, Bes-Rastrollo MM, Fernendez-Crehuet J, Marti A (2010). A 3-year intervention with a Mediterranean diet modified the association between the rs9939609 gene variant in FTO and body weight changes. Int J Obes (Lond).

[31] Roswall N, Ängquist L, Ahluwalia TS, Romaguera D, Larsen SC, Østergaard JN, Halkjær J, Vimaleswaran KS, Wareham NJ, Bendinelli B, Palli D, Boer JMA, van der A DL, Boeing H, Loos RJF, Sørensen TIA, Tjønneland A (2014). Association between Mediterranean and Nordic diet scores and changes in weight and waist circumference: influence of FTO and TCF7L2 loci. Am J Clin Nutr.

